# Sexual Dimorphic Innate Immune Response to a Viral–Bacterial Respiratory Disease Challenge in Beef Calves

**DOI:** 10.3390/vetsci9120696

**Published:** 2022-12-15

**Authors:** Nicole C. Burdick Sanchez, Paul R. Broadway, Jeffery A. Carroll

**Affiliations:** Livestock Issues Research Unit, ARS, USDA Lubbock, Lubbock, TX 79403, USA

**Keywords:** bovine respiratory disease, cattle, cytokines, innate immunity, sexual dimorphism, white blood cells

## Abstract

**Simple Summary:**

Bovine respiratory disease is the leading cause of morbidity and mortality in cattle and is estimated to result in USD 800 to USD 900 million in losses each year. Unfortunately, few improvements have been made over the past 30 years to reduce the negative effects associated with bovine respiratory disease. Many naturally occurring factors, such as breed and temperament, have previously been observed to influence immune responses to infection. However, there is limited information on the influence of sex on the immune response to bovine respiratory disease. Thus, a study was conducted where castrated male (steer) and intact female (heifer) calves were administered a dual viral–bacterial respiratory disease challenge. In response to the challenge, heifer and steer calves produced distinct innate immune responses where steers produced a stronger early acute phase response, while heifers appeared to have a delayed response to the challenge. This suggests heifers may be more resilient to the viral–bacterial challenge when compared with steers. Therefore, the sexually dimorphic immune response should be considered when monitoring cattle for symptoms of bovine respiratory disease

**Abstract:**

The potential for sexually dimorphic innate immune responses to respiratory disease was evaluated, where eight steers and seven heifers (280 ± 4 kg) were subjected to a viral–bacterial respiratory disease challenge utilizing bovine herpesvirus-1 (BoHV-1; intranasal; 1 × 10^8^ PFU/nostril) and *Mannheimia haemolytica* (MH; intratracheal; 1.3 × 10^7^ CFU/head) administered 72 h later. Body temperature was lesser in heifers than steers (*p* < 0.01). There was a sex × time interaction (*p* = 0.05) for white blood cells where heifers had reduced concentrations compared with steers at −72 and 0 h but greater concentrations from 36 to 60 h post-MH. Concentrations of neutrophils were lesser in heifers compared to steers from 0 to 4 h, and from 8 to 12 h (*p* = 0.03). Lymphocytes were greater in heifers compared to steers at 12 h and from 36 to 60 h post-MH (*p* < 0.01). The neutrophil–lymphocyte ratio was lesser in heifers compared to steers from 2 to 24 h and at 48 h post-MH (*p* < 0.01). Monocytes were greater in heifers compared to steers from 24 to 60 h post-MH (*p* < 0.01), while eosinophils were greater in heifers compared to steers at 48 and 60 h (*p* < 0.01). Serum IL-4 was lesser in heifers compared to steers at 0 h and from 2 to 72 h post-MH challenge (*p* = 0.02). Non-esterified fatty acid concentrations were lesser (*p* < 0.01) in heifers compared to steers from 2 to 4 h post-MH challenge. Urea nitrogen concentrations were greater (*p* < 0.01) in heifers than steers at 36 h post-MH challenge. Data from this study reveal distinct differences in the acute phase response following a respiratory disease challenge where steers produced an early response, while the response in heifers appeared to be delayed.

## 1. Introduction

Sexually dimorphic biological responses are not a novel observation, as clear sexual dimorphic phenotypes exist in nature [1]. This can be seen in the mane of a male lion and the elaborate tail feathers of a male peacock. However, sexual dimorphism is much more complex than outward appearance. There is evidence that sexual dimorphism influences neuron signaling in the brain, stress responsiveness, and immunity, as well as many other body systems [2,3,4]. Our laboratory has studied sexual dimorphism in cattle and swine as it relates to stress and immune responsivity. Specifically, cattle exhibit sexually dimorphic responses to activation of the stress axis via corticotropin-releasing hormone [5] and activation of the immune axis with lipopolysaccharide (LPS) [6]. Additionally, a sexually dimorphic immune response to *Salmonella* Typhimurium has been observed in weaned pigs, but there was no effect of sex on tissue *Salmonella* counts following oral challenge [7].

Bovine respiratory disease (BRD) is the leading cause of morbidity and mortality in cattle [8] and is estimated to result in USD 800 to USD 900 million in losses each year [9]. These losses not only include mortality but also reduced performance, lost carcass merit caused by BRD morbidity, and treatment cost. Adding to the difficulty is the fact that BRD is often a multi-pathogen disease, making it difficult to treat and protect cattle against. Therefore, few improvements have been made over the past 30 years to reduce the negative effects on cattle performance and well-being associated with BRD infections. In order to study BRD, we developed a dual challenge viral–bacterial model for beef calves, which consists of an initial intranasal viral challenge with bovine herpesvirus-1 (BoHV-1) followed by an intratracheal challenge with *Mannheimia haemolytica* (MH). This challenge model has been demonstrated to mimic the pathogenesis of a typical viral–bacterial BRD infection and allows for the study of factors that may influence how cattle respond to the disease [10]. Whereas factors such as breed [11] and management practices [12] have been studied, there are limited studies available on the influence of sex on the immune response to BRD [13]. Thus, this study was designed to evaluate a potential sexually dimorphic innate immune response to a respiratory disease challenge in calves.

## 2. Materials and Methods

All experimental procedures were in compliance with the *Guide for the Care and Use of Agricultural Animals in Research and Teaching* and approved by the Institutional Animal Care and Use Committee at the Livestock Issues Research Unit (Protocol # 1606F).

### 2.1. Experimental Design

Crossbred calves approximately 10 months of age (n = 7 intact females (heifers; 267 ± 7 kg) and 8 castrated males (steers; 288 ± 9 kg)) that were selected based on body weight, phenotype, temperament, health status, and serum BoHV-1 titers measured 2 weeks prior to challenge were used for this study. All calves were vaccinated against respiratory pathogens at 21 d before and at weaning using a combination 5-way respiratory modified-live virus vaccine. No *Mannheimia haemolytica* bacterin was administered. Calves were transported approximately 1300 km (12 h) and upon arrival were housed in covered outdoor pens until processed where they had access to hay, a pelleted standard ration and water.

At −72 h (d −3), calves were worked through an indoor working facility and were weighed, fitted with indwelling rectal [14] or vaginal [15] temperature recording devices. Temperature devices were programed to measure body temperature every 5 min for the duration of the study. Additionally, two blood samples were collected via jugular venipuncture for serum isolation and for measurement of complete blood counts. Specifically, a 9 mL vacutainer with no additive was collected and allowed to clot at room temperature for 30 min when it was centrifuged at 1500× *g* for 20 min at 4 °C. Triplicate aliquots were stored at −80 °C until analyzed for cytokines (interleukin-4 (IL-4), IL-6, and interferon-γ (IFN-γ)), haptoglobin, glucose, BoHV-1 titers, non-esterified fatty acids (NEFA), and serum urea nitrogen (SUN). The second sample collected for hematology analysis was collected into a 4 mL vacutainer containing EDTA and was analyzed using the ProCyte Dx Hematology Analyzer (IDEXX Laboratories, Inc., Westbrook, ME, USA) using bovine-specific analogs. Following placement of the temperature devices and collection of blood samples, a visual nasal lesion score was recorded (described below). Lastly, calves were dosed intra-nasally with 1 mL per nostril of 1 × 10^8^ pfu of BoHV-1 using an atomizer device (Teleflex, Morrisville, NC, USA) attached to a 3 mL syringe. Following administration of BoHV-1, calves were returned to the outside pens for 72 h. During this time calves were visually appraised for health daily as described below. 

After 72 h (d 0), calves were processed again through the indoor working facility. Each calf was weighed, fitted with an indwelling jugular vein catheter for collection of serial blood samples, and two blood samples were collected from the cannula for isolation of serum and measurement of hematology variables as previously described. Additionally, a nasal lesion score was recorded. Next, 1.3 × 10^7^ cfu MH was administered in 100 mL of PBS intratracheally. Immediately following these procedures, calves were moved into individual bleeding stalls in an environmentally controlled barn. Serial blood samples for serum isolation were collected into 10 mL Sarstedt tubes containing no additive (Sarstedt, Inc. Newton, NC, USA) via the indwelling cannulas at 0 (immediately prior to MH administration), 1, 2, 3, 4, 5, 6, 7, 8, 12, 24, 36, 48, 60, and 72 h. Additionally, serial blood samples for hematology were collected into 4 mL vacutainers containing EDTA at 0, 2, 4, 6, 8, 12, 24, 36, 48, 60, and 72 h. These samples were processed as described above. While in the stanchions, calves were fed twice daily to maintain ad libitum feeding and had ad libitum access to water provided through paddle-type individual waterers (Suevia Cup; QC Supply Inc., Schuyler, NE, USA) that were connected to a custom-built water intake monitoring system that allowed for the measurement of individual daily water intake as well as water intake bouts.

Following collection of the 72 h sample, steers were worked through the working facility where they were weighed, and temperature devices and jugular cannulas were removed. Additionally, a nasal lesion score was recorded. Lastly, calves were treated with a florfenicol (Nuflor, Merck Animal Health, Madison, NJ, USA) according to label instructions. Following these procedures calves were returned to the outdoor pens. Prior to shipment of cattle to a collaborator, cattle were processed through the working facility on d 7 for collection of a blood sample for BoHV-1 titers and recording of nasal lesion score.

### 2.2. Health and Nasal Lesion Scores

Health scores were assigned to each calf daily based on the adaptation of the system developed by the School of Veterinary Medicine, University of Wisconsin. Calf respiratory scoring chart (available at www.vetmed.wisc.edu/dms/fapm/fapmtools/8calf/calf_respiratory_scoring_chart.pdf, accessed on 01 October 2017). Scoring was performed by 2 trained observers throughout the study. Nasal lesion scores were assigned to calves on days 0, 3, and 7 of the challenge based on a score of 0 to 4 relative to the amount of naris covered by lesions. Specifically, 0 = no visible lesions within the naris, 1 = presence of lesions on < 10% of naris, 2 = presence of lesions on 11 to 25% of naris, 3 = presence of lesions on 26 to 50 percent of naris, and 4 = presence of lesions on >50% of the visible mucosa in the naris.

### 2.3. Serum Analysis

All serum analyses were performed in duplicate. Serum BoHV-1 titers were determined by the Texas Veterinary Medical Diagnostic Laboratory in Amarillo, TX, USA. Concentrations of serum cytokines (IL-4, IFN-γ, and IL-6) were measured by custom bovine 3-plex sandwich-based chemiluminescence ELISA kits (Searchlight-Aushon BioSystems, Inc., Billerica, MA, USA). The minimum detectable concentrations were 2.6, 0.1, and 3.3 pg/mL for IL-4, IFN-γ, and IL-6, respectively. All intra-assay coefficients of variation were less than 6.2% and all inter-assay coefficients of variation were less than 11.8% for all assays.

Glucose concentrations were determined by modification of the enzymatic Autokit Glucose (Wako Diagnostics, Richmond, VA, USA) to fit a 96-well format as previously described [16]. The intra- and inter-assay coefficients of variation were less than 6.4% and 16.2%, respectively. 

Concentrations of NEFA were determined by modification of the enzymatic HR Series NEFA-HR (2) assay (Wako Diagnostics) to fit a 96-well format as previously described [16]. The intra- and inter-assay coefficients of variation were less than 6.1% and 9.5%, respectively.

Concentrations of SUN were determined according to the manufacturer’s directions using a colorimetric assay (K024-H1; Arbor Assays, Ann Arbor, MI, USA). The intra- and inter-assay coefficients of variation were less than 2.1% and 6.9%, respectively.

Serum haptoglobin concentrations were determined using a commercially available ELISA kit according to the manufacturer’s directions (Immunology Consultants Laboratory, Inc. Portland, OR, USA). Concentrations of haptoglobin were determined by comparing unknown samples to a standard curve of known haptoglobin concentrations. The intra- and inter-assay coefficients of variation were less than 4.2 and 3.1%, respectively.

### 2.4. Statistical Analysis

Health, serum BoHV-1 titers, nasal lesion scores, and water intake bouts were analyzed by PROC GLIMMIX with sex, day, and sex × day included as main effects, day included as a random effect, and pen as the subject. Temperature data were averaged into 1 h intervals prior to being analyzed. Temperature, water intake volume, hematology, and serum analyses were analyzed as repeated measure over time with the use of the MIXED procedure of SAS (SAS Inst. Inc., Cary, NC, USA, v. 9.4). Sex, time, and the sex × time interaction were included as fixed effects with calf within sex included as the experimental unit. Covariance structure used was based on having the lowest AICC fit statistic value. When main effects were significant, means were separated using the PDIFF option in SAS, with *p* ≤ 0.05 considered significant and trends toward significance were considered at 0.05 ≤ *p* ≤ 0.10. All data are presented as the LSM ± SEM.

## 3. Results

### 3.1. Health and Nasal Lesion Scores, Body Temperature, and Water Intake

There was a sex × day interaction (*p* = 0.02) for health scores. Specifically, heifers had greater average health scores on days −2, −1, and 1 compared to steers (data not shown). Nasal lesion scores were not influenced by sex (*p* = 0.66; 2.36 ± 0.18 vs. 2.47 ± 0.17 for heifers and steers, respectively) or a sex × time interaction (*p* = 0.39), but they changed over time (day; *p* < 0.01). Specifically, scores increased from d 0 to day 3 (2.19 ± 0.19 vs. 3.13 ± 0.22), before decreasing on d 7 (1.93 ± 0.22). Pre-screen BoHV-1 shedding analysis from nasal swabs was negative for all calves, while all calves were positive for BoHV-1 shedding on d 0 and 3, with the exception of one negative heifer on d 0. Additionally, there was no effect of sex (*p* = 0.22) or a sex × time interaction (*p* = 0.44) for serum BoHV-1 titers, although values changed over time (*p* < 0.01; Table 1).

**Figure 1:**
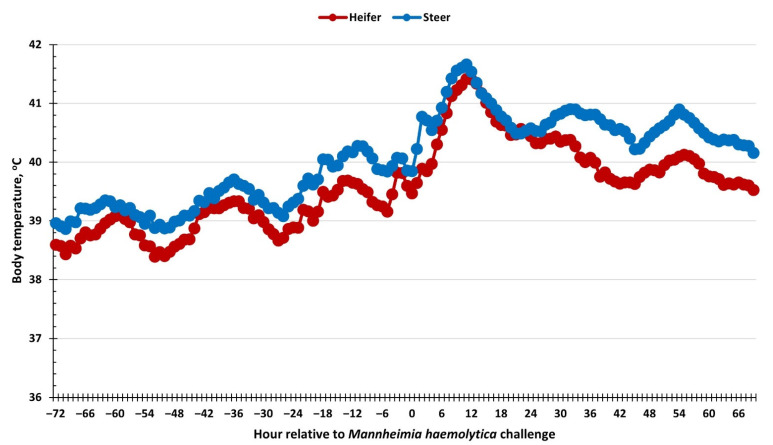
Influence of calf sex on the body temperature response to a dual viral–(bovine herpesvirus-1; 1 × 10^8^ pfu/nostril) bacterial (*Mannheimia haemolytica*; 1.3 × 10^7^ cfu) respiratory disease challenge. Rectal temperature was measured every 5 min and averaged across hours from −72 to 69 h relative to the challenge with *Mannheimia haemolytica*. Body temperature increased (*p* < 0.01) following *Mannheimia haemolytica* challenge and was reduced in heifers compared to steers (sex: *p* < 0.01). SEM ± 0.22 °C.

There was an effect of sex (*p* < 0.01) on body temperature measurements, where heifers had reduced body temperature compared to steers (39.6 vs. 40.1 ± 0.1 °C; Figure 1). Additionally, body temperatures increased overtime (*p* < 0.01). Specifically, temperature increased gradually following the BoHV-1 challenge, with a more robust increase within 6 to 12 h following the MH challenge. There was no sex × time interaction (*p* = 0.54) for body temperature.

There was a tendency (*p* = 0.06) for a sex × time effect for mL of water consumed per hour (data not shown). Specifically, steers consumer greater amounts of water than heifers at 10, 21, 30, 54, and 56 h relative to MH challenge. Additionally, water intake per hour changed over time (*p* < 0.01), but there was no main effect of sex (*p* = 0.27). The number of water intake bouts was similarly affected by time (*p* < 0.01), but there was no effect of sex (*p* = 0.17) or a sex × time interaction (*p* = 0.65).

### 3.2. Hematology

**Figure 2:**
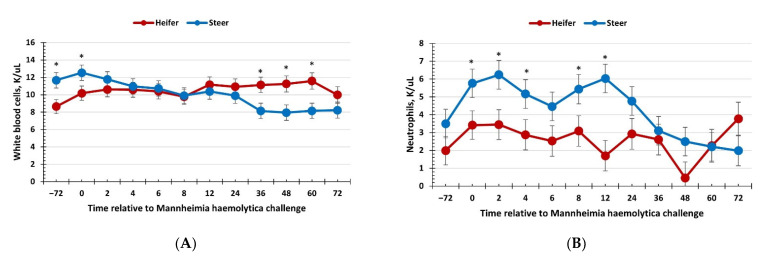

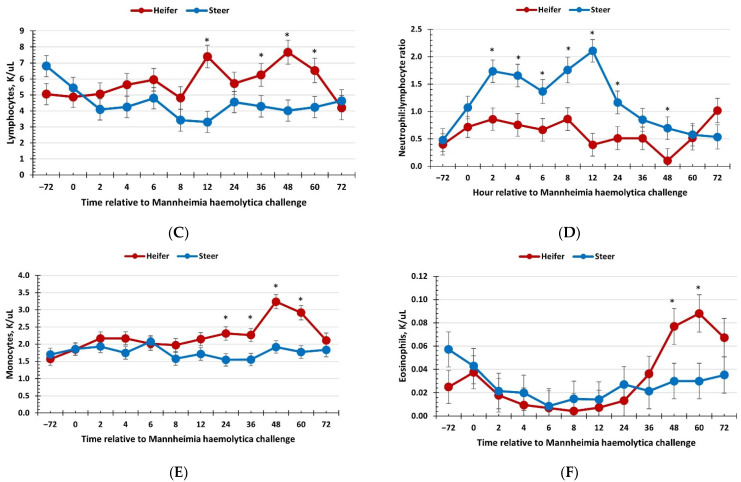
Influence of calf sex on the hematology response to a dual viral–(bovine herpesvirus-1; 1 × 10^8^ pfu/nostril) bacterial (*Mannheimia haemolytica*; 1.3 × 10^7^ cfu) respiratory disease challenge. (**A**) White blood cells: there was a sex × time interaction (*p* = 0.05) where heifers had less white blood cells at −72 and 0 h but had greater concentrations at 36, 48, and 60 h compared to steers. (**B**) Neutrophils were reduced in heifers at 0, 2, 4, 8, and 12 h compared to steers (sex × time: *p* = 0.03). (**C**) Lymphocytes were greater in heifers compared to steers at 12, 36, 48, and 60 h post-challenge (sex × time: *p* < 0.01). (**D**) The neutrophil:lymphocyte ratio was reduced in heifers from 2 to 24 h and at 48 h post-challenge (sex × time: *p* < 0.01). (**E**) Monocytes were greater in heifers from 24 to 60 h post-MH challenge (sex × time: *p* < 0.01) and (**F**) eosinophils were greater in heifers than steers at 48 and 60 h post-challenge (sex × time: *p* < 0.01). * Sexes differ *p* ≤ 0.05.

There was no effect of sex (*p* ≥ 0.34) on total red blood cells, hemoglobin, hematocrit, or platelet concentrations; however, these variables changed over time (*p* < 0.01; Table 2). There was a sex × time interaction (*p* = 0.05) for total white blood cell concentrations, where heifers had reduced concentrations compared with steers at −72 and 0 h (immediately prior to administration of BoHV-1) yet had greater white blood cell concentrations than steers at 36, 48, and 60 h post-MH challenge (Figure 2a). Additionally, there was a sex × time interaction (*p* = 0.03) for neutrophil concentrations, where heifers had reduced concentrations compared with steers at 0, 2, 4, 8, and 12 h post-MH challenge, and tended (*p* = 0.09) to have greater neutrophil concentrations at 48 h post-MH challenge (Figure 2b). Lymphocyte concentrations also exhibited a sex × time interaction (*p* < 0.01), with heifers having greater lymphocyte concentrations than steers at 12, 36, 48, and 60 h post-MH challenge, and heifers tended (*p* ≤ 0.06) to have reduced lymphocyte concentrations at −72 h post-MH challenge (Figure 2c). As expected, based on neutrophil and lymphocyte concentrations, there was a sex × time interaction (*p* < 0.01) for the neutrophil–lymphocyte ratio (Figure 2d). Specifically, heifers had a reduced neutrophil:lymphocyte ratio from 2 to 24 h and at 48 h post-MH challenge. In addition, there was a sex × time interaction for monocyte concentrations (*p* < 0.01), where values were greater for heifers than steers from 24 to 60 h post-MH challenge (Figure 2e), and a sex × time interaction for eosinophil concentrations (*p* < 0.01), with heifers having greater eosinophils at 48 and 60 h post-MH challenge (Figure 2f).

### 3.3. Serum Cytokines and Haptoglobin

**Figure 3:**
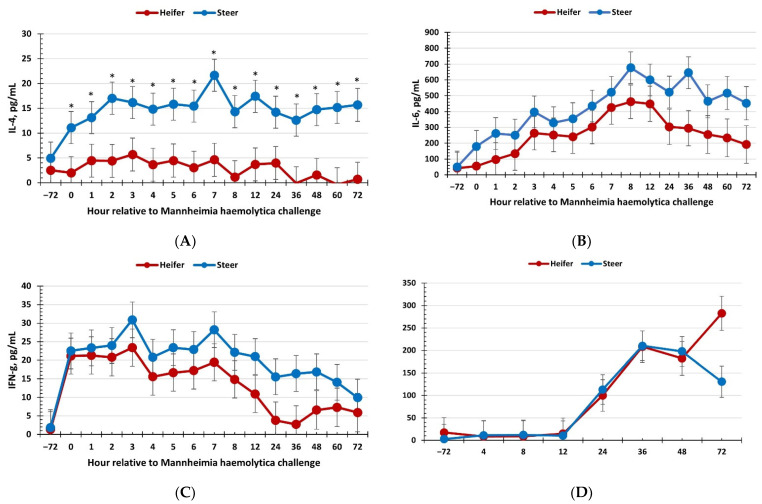
Influence of calf sex on the (**A**) interleukin-4 (IL-4); (**B**) interleukin-6 (IL-6); (**C**) interferon-γ; and (**D**) haptoglobin response to a dual viral–(bovine herpesvirus-1; 1 × 10^8^ pfu/nostril) bacterial (*Mannheimia haemolytica*; 1.3 × 10^7^ cfu) respiratory disease challenge. (**A**) There was a sex × time interaction (*p* = 0.02) for concentrations of IL-4 where they were lower in heifers than steers at 0 h and from 2 to 72 h post-MH challenge. (**B**) There was a tendency (*p* = 0.10) for IL-6 concentrations to be reduced in heifers compared to steers. * Sexes differ *p* ≤ 0.05.

There was a sex × time interaction (*p* = 0.02) for serum concentrations of IL-4, where heifers maintained reduced concentrations of IL-4 concentrations at 0 h and from 2 to 72 h post-MH challenge, with a tendency at 1 h (*p* = 0.06; Figure 3a). Similarly, there was a tendency (*p* = 0.10) for heifers to have reduced IL-6 concentrations compared to steers (Figure 3b). Concentrations of IL-6 increased through approximately 8 h following the MH challenge before decreasing (*p* < 0.01). There was no effect of sex on concentrations of IFN-γ (*p* = 0.27); however, concentrations increased over time (Figure 3c). Specifically, IFN-γ concentrations peaked at approximately 3 h following the MH challenge and gradually decreased over the duration of the challenge. Additionally, there was no effect of sex on the serum haptoglobin response to the respiratory disease challenge (*p* = 0.46; Figure 3d), although concentrations increased from 12 h to 72 h (time: *p* < 0.01).

### 3.4. Serum Glucose, Non-Esterified Fatty Acids, and Urea Nitrogen

**Figure 4:**
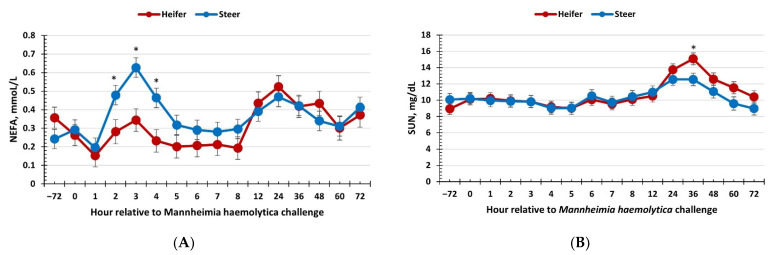
Influence of calf sex on the (**A**) non-esterified fatty acid (NEFA) and (**B**) serum urea nitrogen response to a dual viral–(bovine herpesvirus-1; 1 × 10^8^ pfu/nostril) bacterial (*Mannheimia haemolytica*; 1.3 × 10^7^ cfu) respiratory disease challenge. (**A**) There was a sex × time interaction (*p* < 0.01) for NEFA concentrations where heifers had reduced concentrations from 2 to 4 h post-challenge relative to steers. (**B**) A sex × time interaction (*p* < 0.01) was observed for serum urea nitrogen concentrations such that heifers had greater concentrations at 36 h and tended to have greater concentrations at 60 h compared to steers. * Sexes differ *p* ≤ 0.02.

There was no effect of sex on the serum glucose response to the respiratory disease challenge (*p* = 0.14). However, concentrations of glucose decreased over time (*p* < 0.01), with greater concentrations observed from −72 to approximately 4 h following the MH challenge before decreasing. A sex × time interaction (*p* < 0.01) was observed for serum NEFA concentrations, where heifers had reduced concentrations of NEFA compared to steers from 2 to 4 h post-MH challenge (Figure 4a). Additionally, there was a sex × time interaction (*p* < 0.01) for SUN concentrations, where heifers had greater SUN concentrations at 36 h and tended (*p* = 0.08) to have greater SUN concentrations at 60 h post-MH challenge (Figure 4b).

## 4. Discussion

The multifaceted nature of BRD makes it a difficult disease to study due to the many viral and bacterial agents associated with BRD pathogenesis. Both BoHV-1 and MH are two of the most common pathogens associated with BRD, thus making this model applicable for the study of the disease [17]. Body temperature is typically measured when a morbid calf is brought out of a pen for a health evaluation. Differences in body temperature have been observed in response to immune challenges due to natural variations such as breed, temperament, and sex [6,18,19]. This appears to be one of the first studies to find differences in body temperature between heifers and steers in response to a respiratory disease challenge. The greater body temperature exhibited by steer relative to heifer calves coincides with the tendency for greater concentrations of IL-6, a pro-inflammatory cytokine often associated with increasing body temperature in response to an immune challenge [20,21]. While heifers had reduced average body temperature compared to steers, it appears both sexes produced a similar response profile to the challenge as there was no sex × time interaction. Reduced body temperature in male compared to female mice in response to a *Streptococcus pneumoniae* infection has been observed [22]. Similarly, body temperature was reported to be greater in heifers compared to steers challenged with lipopolysaccharide [6]. While the decreased body temperature response appears to be detrimental to the survival of male mice, the reduced body temperature response in steers in response to LPS may suggest a stronger innate immune response in steers. Both of these aforementioned studies contrast with the current study where body temperature was greater in steers than heifers. The reduced body temperature in heifers may be associated with the reduced breakdown of energy sources. While no differences were observed in glucose concentrations, heifers had reduced concentrations of NEFA in the early hours following the MH challenge, which suggests that heifers did not need to break down adipose tissue for energy or could point towards greater insulin concentrations [23]. In contrast, the greater body temperature in steers could be associated with greater feed intake, as steers were heavier than heifers at the onset of the study. Thus, the metabolic implication of reduced body temperature in heifers relative to steers requires additional study.

While all cattle were vaccinated using the same multivalent vaccine against common respiratory disease viruses, all calves exhibited a response to the challenge. Serum samples collected approximately two weeks prior to the study were used to balance calves by BoHV-1 titers, and thus these did not differ between heifers and steers prior to the start of the study. Serum BoHV-1 titers increased throughout the study, but there was no effect of sex or sex × time interaction. However, it is important to note that there was a large numerical difference in BoHV-1 titers between heifers and steers on d 7, which was 10 days following administration of the intranasal BoHV-1. Data in other species have found that females have a greater adaptive immune response (greater number of B lymphocytes, greater immunoglobulin concentrations) compared to males [1,24,25]. Additionally, the literature suggests a weaker vaccine response in males compared to females [26,27]. The lack of statistical significance may be due to the number of cattle utilized in the study as well as the high amount of variation in the IBR titer data. Thus, it is possible that a poorer response to BRD viral vaccination in steer calves contributed to the greater acute phase response observed and should be studied in greater detail. Additionally, it is possible that the cattle naturally carried other respiratory pathogens that were not tested for, such as bovine viral diarrhea virus.

The hematological response between heifers and steers was very interesting, with significant differences observed in the WBC differential counts. Neutrophil concentrations increased in steers following the challenge with BoHV-1, as indicated by the greater concentrations at 0 h. In contrast, neutrophil concentrations appeared to begin increasing later in the challenge period in heifers. Thus, there appeared to be a quicker neutrophil response by the steers, suggesting steers may have been more sensitive to the challenge compared to heifers, which displayed a later response. In future studies, it would be interesting to extend the sampling period to see if neutrophil concentrations continue to increase in heifers following the 72-h sample. An increase in neutrophils in response to BRD infection has been previously reported [28]. Additionally, it has been reported that BoHV-1 can cause immunosuppression and reduce the migration of neutrophils [29,30], which may explain the greater neutrophils in steers compared to heifers in the current study. In contrast to neutrophils, lymphocyte concentrations increased in heifers following the MH challenge, while lymphocyte concentrations decreased in steers. Future studies should determine if certain subsets of lymphocytes are more prevalent during BRD infection, and how these populations are influenced by sex. The lymphocyte response together with neutrophil concentrations, explains the reduced neutrophil:lymphocyte ratio in heifers. The neutrophil–lymphocyte ratio can be used as an indicator of increased inflammation. Neutrophils are contributors of inflammation within tissue, and greater concentrations of neutrophils, indicated by a neutrophil:lymphocyte ratio greater than 1, may be indicative of greater tissue inflammation. Thus, this suggests steers produced a greater inflammatory response to the BRD challenge when compared to heifers. An increase in neutrophil concentrations and the neutrophil:lymphocyte ratio have been associated with naturally occurring BRD infection [31]. The greater lymphocyte concentrations in heifers may be suggestive of a greater adaptive immune response [1,27], or could reflect an increase in either diapedesis or leukocytosis in steers [32]. On the other hand, the greater neutrophil response is suggestive of more severe disease in steers and is supported by the greater concentrations of pro-inflammatory cytokines. Studies in a variety of species have demonstrated a greater inflammatory response of males to endotoxin challenge [6,27], and data from the current study perhaps reflect the greater response to the endotoxin or leukotoxin associated with MH challenge. In fact, rodent models have found greater expression of Toll-like Receptor 4 (TLR4), which recognizes endotoxin, on immune cells isolated from males [33]. The greater monocyte and eosinophil concentrations observed in heifers are interesting as they occurred much later relative to the challenge with MH, further supporting a delayed immune response in heifers. It is possible that the later increase in monocytes represents activation of the adaptive immune response, as activated monocytes (macrophages) are one of the major antigen-presenting cells responsible for activating effector functions of lymphocytes, which also exhibited a similar temporal pattern in heifers. On the other hand, an increase in monocytes and thus activated macrophages may be necessary for the clearance of tissue debris within the lungs as disease progresses. There is evidence for a role of eosinophils in modulating immune responses, specifically with lymphocytes, and thus the increase in eosinophils in heifers may be related to the similar rise in lymphocytes [34,35]. Furthermore, Leach, Chitko-McKown, Bennet, Jones, Kachman, Keele, Leymaster, Thallman, and Kuehn [34] reported that cattle with greater eosinophils required treatment for BRD less often. This is supported by Richeson et al. [36] who reported that low eosinophils and high red blood cells may be used to identify cattle at greater risk of BRD development. Overall, the WBC differential counts support the conclusion that steers produced a greater inflammatory response early in response to the challenge, while the response from the heifers appeared delayed.

The responses observed for cytokines, while not all statistically significant, further support a greater inflammatory response by steers. As discussed above, greater TLR4 expression on immune cells isolated from male rodents may be a driving factor associated with the greater pro-inflammatory cytokine response [33]. Indeed, greater concentrations of pro-inflammatory cytokines have been associated with greater susceptibility to septic shock induced by endotoxin [27]. It is highly probable that the reduced production of cytokines by heifer calves is directly related to the reduced number of leukocytes observed in circulation. Production of IL-4 is associated with the activation of CD4+ T helper cells and B cells as well as alternatively activated macrophages (M2), and thus plays a more anti-inflammatory role [37]. There appeared to be no increase in IL-4 concentrations in heifers, while concentrations in steers were on average more than 5-fold greater compared to heifers. The increase in IL-4 in steers may be a result of an increased inflammatory response resulting in a greater need for increased anti-inflammatory cytokines to regulate the immune response. However, further research is needed in this area. As discussed earlier, IL-6 is responsible for increasing body temperature in response to pathogen exposure and has roles in stimulating sickness behaviors and activating acute phase protein production in the liver [38,39,40]. The tendency for lesser IL-6 concentrations in heifers compared to steers is supportive of the lesser body temperature response observed in heifers. A study in calves challenged with bovine viral diarrhea virus and/or MH observed an increase in IL-6 concentrations in response to the challenge, similar to that observed in the current study [41]. Similar to the current study, a study in humans found that isolated peripheral blood mononuclear cells from males challenged with LPS produced more IL-6 than cells isolated from females [42]. Thus, while differences in IL-6 concentrations in the current study only tended to be different, there is support in the literature suggesting an effect of sex on the IL-6 response to immune challenges. Interferon-γ is important for the antiviral response to infection, and thus plays an important role in BRD infections that are often associated with a viral component. Specifically in response to BoHV-1, T-lymphocytes secrete IFN-γ to stimulate lysis of virus-infected cells [43]. Thus, the increase in IFN-γ concentrations three days following BoHV-1 infection was expected and is in line with previously reported studies [44]. While values for IFN-γ were not statistically different between heifers and steers, they did trend in a similar manner to what was observed for IL-4 and IL-6. Therefore, the cytokine response supports an increase in inflammation in steers compared to heifers and is in line with results observed in both body temperature and leukocyte concentrations.

Acute phase proteins are important for combating both viral and bacterial infections. In particular, haptoglobin has a role in reducing iron availability to bacteria through binding and sequestering hemoglobin [45]. The literature has reported an association between haptoglobin and BRD in cattle [46,47]. Godson et al. [48] reported increases in haptoglobin in beef calves challenged with BoHV-1 and *Pasteurella haemolytica*. While there was no effect of sex or a significant sex × time interaction for the haptoglobin response in the current study, it is interesting to note the marked numerical difference between heifers and steers at 72 h, where haptoglobin appears to remain elevated in heifers, yet concentrations in steers appear to be decreasing. This would further support the theory of a quicker response from the steers resulting in recovery, yet a later or delayed response in the heifers. Thus, this aspect of the study bears repeating to determine if a relationship exists between sex and haptoglobin concentrations in response to a BRD challenge, as haptoglobin has been reported as one variable that can be used as a BRD diagnostic tool [49].

Available energy sources are extremely important for immune activation, the acute phase response, and recovery from an infection. A study by Kvidera et al. [50] found that cattle may use over 1 kg of glucose within 12 h after being challenged with LPS. Failure to have adequate energy may limit the ability of the immune system to fight off a pathogen, thus extending the duration of an immune challenge or worsening the negative effects [51]. Compounding the need for suitable energy resources is the fact that calves typically reduce feed intake in response to an infection [52,53]. Thus, maintaining adequate energy stores prior to exposure to pathogens is vital to the ability of a calf to respond to and recover from an infectious agent in a quick and controlled manner. In response to the BRD challenge, there was no difference observed in glucose concentrations between heifers and steers. This was interesting as glucose is the primary fuel for immune cells. In contrast to glucose, there was a reduced NEFA response early post-challenge (2 to 4 h) in heifers compared to steers. It can be argued that this difference may be due to differences in adiposity, as steers weighed more than heifers at the onset of the study. Considering the differences observed in the other variables measured, the greater NEFA concentrations suggest that steers may have required a greater amount of energy than could be provided by glucose alone, thus resulting in the breakdown of adipose tissue. The greater SUN concentrations observed in heifers later in the challenge support the delayed immune response in heifers. On the other hand, the increase in SUN may also be associated with an increase in protein degradation to provide amino acids for various immune system responses, such as the production of acute phase proteins. However, no differences in haptoglobin concentrations were observed between sexes. Nonetheless, the differences in SUN concentrations between heifers and steers were minimal and may not be biologically significant. 

## 5. Conclusions

Distinct differences exist in the acute phase response of heifers and steers to a viral–bacterial respiratory disease challenge. Steers produced an early response to the challenge, as indicated by the greater neutrophil, IL-4, and NEFA response, while the response from the heifers appeared later or delayed based on lymphocyte, monocyte, eosinophil, and SUN values. This suggests that the heifers may have been more resilient than steers and required a lesser inflammatory response to the challenge. However, a strong immune challenge that overwhelms the innate immune response may require medical intervention in heifers similar to steers, and these data are not indicative of the response of calves to therapeutic treatment for BRD. Thus, the sexually dimorphic immune response to respiratory disease should be considered when monitoring calves for symptoms of BRD.

## Figures and Tables

**Table 1:** Bovine herpesvirus-1 (BoHV-1) serum titers in heifers and steers exposed to a dual viral–bacterial respiratory disease challenge.

					*p*-Value		
Variable	Day	Heifer	Steer	SEM	Sex	Time	Sex × Time
BoHV-1 titers							
	Pre-screen	5.5	5.7	16.9			
	d −3	6.5	4.0	16.9			
	d 0	12.7	4.6	18.2			
	d 7	104.5	56.0	18.2	0.22	<0.01	0.44

**Table 2:** Influence of sex on the complete blood count response to a dual viral–bacterial respiratory disease challenge.

				*p*-Value		
Variable	Heifer	Steer	SEM	Sex	Time	Sex × Time
Red blood cells, M/µL	8.64	8.48	0.56	0.83	<0.01	0.55
Hemoglobin, g/dL	10.90	10.77	0.28	0.73	<0.01	0.49
Hematocrit, %	34.29	34.39	0.96	0.93	<0.01	0.48
Platelets, K/µL	516.84	456.21	38.13	0.27	<0.01	0.36
White blood cells, K/µL	10.51	10.02	0.60	0.57	0.02	0.05
Neutrophils, K/µL	2.56	4.26	0.45	0.02	<0.01	0.03
Lymphocytes, K/µL	5.76	4.48	0.35	0.02	0.02	<0.01
Neutrophil:Lymphocyte	0.60	1.17	0.10	<0.01	<0.01	<0.01
Monocytes, K/µL	2.23	1.77	0.09	<0.01	<0.01	0.01
Eosinophils, K/µL	0.03	0.03	0.01	0.74	<0.01	<0.01

## Data Availability

The original contributions presented in this study are in the article. Further inquiries can be directed to the corresponding authors.
